# The Role of the Medial Prefontal Cortex in Self-Agency in Schizophrenia

**DOI:** 10.20900/jpbs.20210017

**Published:** 2021-10-14

**Authors:** Karuna Subramaniam

**Affiliations:** Department of Psychiatry, University of California, San Francisco, CA-94143, USA

**Keywords:** schizophrenia, self-agency, self-predictions, reality monitoring, speech monitoring, medial prefrontal cortex, psychotic symptoms

## Abstract

Schizophrenia is a disorder of the self. In particular, patients show cardinal deficits in self-agency (i.e., the experience and awareness of being the agent of one’s own thoughts and actions) that directly contribute to positive psychotic symptoms of hallucinations and delusions and distort reality monitoring (defined as distinguishing self-generated information from externally-derived information). Predictive coding models suggest that the experience of self-agency results from a minimal prediction error between the predicted sensory consequence of a self-generated action and the actual outcome. In other words, the experience of self-agency is thought to be driven by making reliable predictions about the expected outcomes of one’s own actions. Most of the agency literature has focused on the motor system; here we present a novel viewpoint that examines agency from a different lens using distinct tasks of reality monitoring and speech monitoring. The self-prediction mechanism that leads to self-agency is necessary for reality monitoring in that self-predictions represent a critical precursor for the successful encoding and memory retrieval of one’s own thoughts and actions during reality monitoring to enable accurate self-agency judgments (i.e., accurate identification of self-generated information). This self-prediction mechanism is also critical for speech monitoring where we continually compare auditory feedback (i.e., what we hear ourselves say) with what we expect to hear. Prior research has shown that the medial prefrontal cortex (mPFC) may represent one potential neural substrate of this self-prediction mechanism. Unfortunately, patients with schizophrenia (SZ) show mPFC hypoactivity associated with self-agency impairments on reality and speech monitoring tasks, as well as aberrant mPFC functional connectivity during intrinsic measures of agency during resting states that predicted worsening psychotic symptoms. Causal neurostimulation and neurofeedback techniques can move the frontiers of schizophrenia research into a new era where we implement techniques to manipulate excitability in key neural regions, such as the mPFC, to modulate patients’ reliance on self-prediction mechanisms on distinct tasks of reality and speech monitoring. We hypothesize these findings will show that mPFC provides a unitary basis for self-agency, driven by reliance on self-prediction mechanisms, which will facilitate the development of new targeted treatments in patients with schizophrenia.

## INTRODUCTION

Self-agency is defined as the experience and awareness of being the agent of one’s own thoughts, actions and action outcomes, and provides the founding basis for our interactions with the world (i.e., reality monitoring) [1–5]. Patients with schizophrenia (SZ) show cardinal deficits in self-agency that contribute to distortions in reality monitoring (impairments in distinguishing self-generated from externally-produced events) [6–9]. For example, hallucinations are thought to result from the misattribution of patients’ internal thoughts as external voices; and delusions of influence in schizophrenia occur when patients feel their own actions are no longer controlled by themselves [10]. These psychotic symptoms of hallucinations and delusions are thought to result from impaired self-predictions about the expected outcome of one’s own actions [3,10,11]. Thus, the psychopathology of hallucinations and delusions suggest patients show reduced reliance on self-predictions about their own action outcomes, misattributing them as being externally-produced, which is thought to result in patients’ lost sense of self-agency and break from reality (i.e., impaired reality-monitoring) [3,11].

## ROLE OF THE MEDIAL PREFRONTAL CORTEX IN SELF-AGENCY

We have found a potential neural substrate of this self-prediction ability. We and others have shown that activity within the medial prefrontal cortex (mPFC) mediates both self-predictions during speech monitoring [12], and self-agency judgments during reality monitoring [6,13]. The agency literature has been dominated by a focus on motor acts [14–16]. It is important to take into account behavioral deficits of the specific patient population and delineate causal biomarkers of the underlying neural aberrations that induce these behavioral self-agency deficits in order to develop new effective treatments. With this goal in mind, given that SZ manifest auditory hallucinations, which are thought to result from impaired self-prediction mechanisms of speech monitoring that result in the misattribution of their inner speech as an external voice [3,11,17,18], we integrate prior findings using distinct reality monitoring and speech monitoring tasks as valid and relevant paradigms for assaying mPFC modulation of self-agency that provides a novel viewpoint and unitary basis for self-agency, driven by the reliance on self-prediction mechanisms in SZ.

## MPFC MODULATES SELF-PREDICTION MECHANISMS THAT UNDERLIE SELF-AGENCY DURING REALITY MONITORING TASKS

Self-agency is a necessary component of reality monitoring, and is thought to depend on making reliable predictions about the expected outcomes of one’s own actions [19,20]. This self-prediction mechanism is a critical precursor for the successful encoding and memory retrieval of one’s own thoughts and actions during reality monitoring to enable accurate self-agency judgments (i.e., accurate identification of self-generated information) [21]. We have found that the medial prefrontal cortex (mPFC) is a potential neural substrate of this self-prediction mechanism [6,21]. The mPFC is one critical region that replicably shows increased activity prior to self-generated actions (that does not occur before externally-perceived actions), and is thought to mediate the consistent preparatory signal that enables and leads to self-agency, shown across convergent evidence from imaging studies (functional MRI, magnetoencephalography (MEG) and electroencephalography (EEG)) and single neuron studies [6,21–27]. In our reality monitoring task, in which subjects distinguish self-generated from externally-derived information, healthy controls (HC) showed increased mPFC activity, shown in beta power suppression, that was observed within a specific time window preceding the successful encoding and retrieval of self-generated information, which correlated with accurate judgments of self-agency, indicating mPFC represents one neural correlate of the self-prediction mechanisms that leads to self-agency [6,21].

Given these correlative data that mPFC support self-prediction mechanisms that lead to self-agency, in a recent study we implemented repetitive transcranial magnetic stimulation (rTMS) to examine causal mechanisms underlying mPFC function on self-agency during reality-monitoring tasks in HC [22]. We found that high-frequency 10Hz rTMS targeting the mPFC site that mediates self-agency in HC and SZ, significantly improved self-agency judgments, when compared to sham stimulation and baseline assessments in HC [22]. This study establishes the mPFC as a novel brain target that can be stimulated with rTMS to causally impact self-agency on reality-monitoring tasks.

## MPFC MODULATES SELF-PREDICTION MECHANISMS THAT UNDERLIE SELF-AGENCY DURING SPEECH MONITORING TASKS

Self-prediction mechanisms are also critical for speech monitoring where we continually compare auditory feedback (i.e., what we hear ourselves say) with what we expect to hear [20]. Prior studies have also found increased mPFC activity (shown in beta power suppression), during self-predictions in ‘self-generated forward models [28,29] (also known as efference copies/corollary discharge) [26,29–31] of speech monitoring where we continually compare what we hear while we speak with what we expect to hear. Speakers experience self-agency when there is minimal prediction error (i.e., when auditory feedback minimally deviates from predictions of what they expect to hear) [19,20]. When subjects hear experimenter-induced pitch perturbations in their auditory feedback while speaking, they make corrective responses, indicating that they judge the perturbations as errors in their speech output [32–36]. These corrective responses are modulated by subjects’ reliance on self-predictions about their speech outcome; the more they rely on their self-predictions, the less they rely on perturbed external auditory feedback, resulting in smaller corrective responses and an enhanced sense of self-agency that they followed their self-predictions to guide their own speech output [19,20].

To provide support for a unitary sense of self-agency that is driven by reliance on self-predictions, we asked HC to complete a speech monitoring task and a reality monitoring task. This experiment tested whether activity in mPFC modulated subjects’ reliance on efference copy self-prediction mechanisms in a speech monitoring task in order to predict and enhance self-agency judgments in a different reality monitoring task. In the speech monitoring task, we perturbed auditory feedback by +/− 1/12 of an octave while HC vocalized the vowel /a/ while listening to their own speech. We found that subjects who made smaller corrective responses during speech perturbations, also had enhanced self-agency judgments during reality monitoring [20]. In other words, the more subjects relied on their self-predictions about their expected speech outcome, the less they relied on external perturbed auditory feedback, resulting in smaller corrective responses and an enhanced sense of self-agency. HC also showed increased mPFC activity during the reality monitoring task [6], and when they made smaller corrections to pitch perturbations in their auditory feedback, indicating greater reliance on self-predictions of their speech outcome [12]. These recent findings provide support for a unitary sense of self-agency, supported by mPFC activity that modulates ‘the amount of reliance’ that need to be placed on self-predictions to potentiate accurate identification of self-generated information on our reality monitoring task that results in the experience of self-agency.

## SELF-PREDICTION MECHANISMS ARE IMPAIRED IN SCHIZOPHRENIA

Normally during speech monitoring, it is thought that efference copy self-prediction mechanisms suppress the auditory cortical response to self-generated sounds, compared to listening to external speech in HC [34,35,37,38]. Such suppression is thought to be the basis for the capacity to experience self-agency, which allows self-generated speech to be distinguished from externally-derived speech. In other words in HC, self-generated (and therefore highly predictable) sounds give rise to suppressed responses, thus allowing speakers to pay better attention to sounds in the external environment [35,37], indicative of a primordial biological basis for self-agency that is essential for normal interactions with outside reality [37,39,40]. By contrast, in SZ, reduced suppression to self-generated actions (i.e., during speaking, for example) [31,37,41–43] suggests patients may have noisier auditory cortical signal to begin with, making it more difficult to make reliable computations about the comparisons between predicted and actual auditory feedback while speaking. This auditory cortical signal is sent to higher order regions within PFC/mPFC [12,36] that are considered to be critical for computing the reliance that needs to be placed on self-predictions [12], in order to mediate higher-order agency judgments [19,20]. Thus, in SZ mPFC regions need to “work much harder” at generating agency judgments because of a combination of: impaired self-predictions (i.e., impaired efference copy signals) [3,11,37], “noisier” computations that are delivered from auditory cortex during self-generated speech [31,41,42], and because mPFC is hypoactive in SZ [6,13].

Consistent with prior data, it is our view that the impaired reliance on self-predictions (also known as prospective intentional binding) may lead patients to show increased binding and dependencies on external environmental cues, in which patients retrospectively over-associate their actions with subsequent events (retrospective intentional binding) [2,10,44–48]. This impaired reliance on self-predictions may thus lead to an increased tendency to misattribute self-generated thoughts and actions to external agents rather than to oneself, or when an internal prediction is weak the binding of the action to an outcome can occur retrospectively [47,49]. An alternative hypothesis that examines agency within a Bayesian framework is that psychosis may result from overweighting of prior predictions [50]. It is important to disentangle the differences between impaired self-predictions in feed-forward models from “top-down” reliance/confidence in these self-predictions (which our data suggests is supported by mPFC). If this alternative hypothesis is supported, in which self-agency impairments result from an over-reliance on prior predictions, we would expect that after high-frequency rTMS of mPFC, this over-reliance in self-predictions would be observed in mPFC activity increases which would correlate with self-agency impairments and psychotic symptoms. Yet, in the findings from the authors in this study that support this alternative hypothesis, hallucinators showed mPFC hypoactivity compared to non-hallucinators [50], consistent with our data showing this hypoactivity is associated with self-agency deficits [6,13] and with other prior findings [51]. Prior studies also show that the mPFC is a critical region for belief-updating and learning associative inferences [52,53], based on using prior experiences to modulate one’s current state in order to guide future reliable self-predictions [12,26,29] that are fundamental for self-agency judgments. In summary, in our current work, we now examine if mPFC specifically modulates the reliance on self-prediction signals in SZ, in which case rTMS-induced increased mPFC activation (shown in beta band frequencies) will induce smaller corrective responses during speech monitoring that will correlate with improved self-agency judgments during reality monitoring and improved positive symptoms in SZ.

It must also be noted that we are not stating that the mPFC represents the only neural correlate of self-agency. Indeed, prior meta-analyses has shown that other regions (e.g., insula) mediate self-agency while the temporo-parietal junction (TPJ) was shown to mediate external agency or a lack of agency [14,15]. However, these meta-analyses only focused on agency attributions of movement to oneself (self-agency) or externally (external agent) rather than on reality and speech monitoring tasks and have only been completed in healthy controls. It is also important to understand the mechanisms of action underlying agency in SZ, and that different mechanisms may contribute to different types of symptoms (positive, negative, paranoid symptoms) driven by distinct agency attributions just within the SZ population itself [54]. Consistent with another meta-analyses [55], it is our view that the TPJ is a heteromodal site that integrates multimodal information from sensory and motor regions to compute integrative prediction comparisons [34], (i.e., the mismatch between the expected and actual outcome of one’s own actions) [34,35] that precede the resulting judgments of agency. The smaller the mismatch, the more likely the outcome will be attributed to oneself. However, we also believe that self-agency does not directly result from the match between predicted and observed feedback. Rather, we favor the view that these self-agency judgments are formulated between reverberating mPFC inter-modular propagation signals between the mPFC and TPJ [46], which produces an estimation of ‘the amount of reliance’ that needs to be placed on self-predictions and the prediction error [20] in order to mediate higher-order agency judgments [19,20]. In our current work we now implement rTMS to increase mPFC and TPJ excitation to disentangle mechanisms of self vs external agency on MEG reality and speech monitoring tasks from pre-to-post rTMS, and to test the causal impact of increasing activity within these neural sites on agency and psychotic symptoms in SZ. We hypothesize that after rTMS of mPFC, improved self-agency judgments will be driven by improved sensitivity in participants’ reliance on their self-predictions, whereas after rTMS of TPJ, we hypothesize that participants will show increased prediction error sensitivities that will potentiate external-agency judgments.

## CONCLUSIONS

Our research findings suggest that the medial prefrontal cortex (mPFC) mediates the reliance of self-prediction mechanisms that lead to the experience of self-agency [12,20,21]. In our reality monitoring task, in which subjects distinguish self-generated from externally-derived information, HC showed increased mPFC activity preceding the successful encoding and retrieval of self-generated information, which correlated with accurate judgments of self-agency, indicating mPFC is a neural correlate of this self-prediction mechanism that leads to self-agency [6,21]. By contrast, SZ showed mPFC hypoactivity associated with self-agency impairments on reality-monitoring tasks [6,13], as well as aberrant mPFC functional connectivity during intrinsic measures of agency reflected in ongoing self-related processing during resting states that predicted worsening psychotic symptoms [5,56,57]. In our current work, we aim to implement causal neurostimulation tools such as TMS to disentangle mechanisms of self vs external agency on MEG reality and speech monitoring tasks from pre-to-post TMS, as well as to disentangle reliance on self-predictions in feed-forward prospective models from retrospective models [58] in speech perturbation and adaptation experiments to delineate the causal impact of how modulating mPFC activity impacts self-agency and psychotic symptoms in SZ, driven by improved reliance on self-prediction mechanisms.
